# Nucleic Acid-Sensing Toll-Like Receptors Play a Dominant Role in Innate Immune Recognition of Pneumococci

**DOI:** 10.1128/mBio.00415-20

**Published:** 2020-03-24

**Authors:** Agata Famà, Angelina Midiri, Giuseppe Mancuso, Carmelo Biondo, Germana Lentini, Roberta Galbo, Maria Miriam Giardina, Giuseppe Valerio De Gaetano, Letizia Romeo, Giuseppe Teti, Concetta Beninati

**Affiliations:** a Department of Human Pathology, University of Messina, Messina, Italy; b Department of Chemical, Biological and Pharmaceutical Sciences, University of Messina, Messina, Italy; c Charybdis Vaccines Srl, Messina, Italy; d Scylla Biotech Srl, Messina, Italy; National Institute of Allergy and Infectious Diseases

**Keywords:** *Streptococcus pneumoniae*, neutrophils, chemokines, interleukin-12, cytokines, innate immunity, macrophages, Toll-like receptors

## Abstract

Streptococcus pneumoniae (or pneumococcus) is a highly prevalent human pathogen. Toll-like receptors (TLRs) function as immune sensors that can trigger host defenses against this bacterium. Defects in TLR-activated signaling pathways, including deficiency in the adaptor protein myeloid differentiation factor 88 (MyD88), are associated with markedly increased susceptibility to infection. However, the individual MyD88-dependent TLRs predominantly involved in antipneumococcal defenses have not been identified yet. Here we find that triple knockout mice simultaneously lacking TLR7, TLR9, and TLR13, which sense the presence of bacterial DNA (TLR9) and RNA (TLR7 and TLR13) in the phagolysosomes of phagocytic cells, display a phenotype that largely resembles that of MyD88-deficient mice and rapidly succumb to pneumococcal pneumonitis due to defective neutrophil influx into the lung. Accordingly, TLR7/9/13 triple knockout resident alveolar macrophages were largely unable to respond to pneumococci with the production of neutrophil-attracting chemokines and cytokines. Mice with single deficiencies of TLR7, TLR9, or TLR13 showed unaltered ability to control lung infection but were moderately more susceptible to encephalitis, in association with a decreased ability of microglia to mount cytokine responses *in vitro*. Our data point to a dominant, tissue-specific role of nucleic acid-sensing pathways in innate immune recognition of S. pneumoniae and also show that endosomal TLRs are largely capable of compensating for the absence of each other, which seems crucial to prevent pneumococci from escaping immune recognition. These results may be useful to develop novel strategies to treat infections by antibiotic-resistant pneumococci based on stimulation of the innate immune system.

## Abstract (precis)

The pneumococcus is a bacterium that frequently causes infections in the lungs, ears, sinus cavities, and meninges. During these infections, body defenses are triggered by tissue-resident cells that use specialized receptors, such as Toll-like receptors (TLRs), to sense the presence of bacteria. We show here that pneumococci are predominantly detected by TLRs that are located inside intracellular vacuoles, including endosomes, where these receptors can sense the presence of nucleic acids released from ingested bacteria. Mice that simultaneously lacked three of these receptors (specifically, TLR7, TLR9, and TLR13) were extremely susceptible to lung infection and rapidly died after inhalation of pneumococci. Moreover, tissue-resident macrophages from these mice were impaired in their ability to respond to the presence of pneumococci by producing inflammatory mediators capable of recruiting polymorphonuclear leucocytes to infection sites. This information may be useful to develop drugs to treat pneumococcal infections, particularly those caused by antibiotic-resistant strains.

## INTRODUCTION

Streptococcus pneumoniae, or pneumococcus, is a Gram-positive encapsulated bacterium that frequently colonizes the upper respiratory tract of humans. This organism, however, can also behave as a dreadful pathogen, estimated to cause globally millions of deaths per year, particularly in children and in the elderly (1, 2). Pneumococci are the most common cause of bacterial community-acquired pneumonia (3) and are, in addition, frequent agents of otitis media, conjunctivitis, meningitis, and septicemia (1).

All these infections are associated with a vigorous inflammatory response that plays a key role in host defenses. Excessive, prolonged, or generalized inflammation, however, can trigger dangerous complications, such as lung injury and septic shock. Respiratory epithelial cells, alveolar macrophages, and dendritic cells first trigger inflammation upon immune recognition of pneumococci entering the lung. Such recognition is mediated by specific receptors (designated pattern recognition receptors), among which Toll-like receptors (TLRs) play an important role through their ability to detect conserved microbial molecules, such as bacterial lipoproteins and nucleic acids (4). Specialized adaptor proteins, such as MyD88, Mal, TRIF, and TRAM, transduce in the cytosol the signals originated by activation of the TLRs present on the cell membrane or in phagosomes. MyD88 and the downstream protein interleukin-1 receptor-associated kinase 4 (IRAK-4) are involved in signaling from all TLRs, with the exception of TLR3, and from the interleukin-1 (IL-1) family receptors IL-1R and IL-18R. MyD88/IRAK-4 signaling ultimately results in the activation of mitogen-activated protein kinases and of various transcription factors, including nuclear factor κB (NF-κB). This leads to the production of proinflammatory cytokines, such as tumor necrosis factor alpha (TNF-α) and IL-1β, which act in concert with the chemokines Cxcl1 and Cxcl2 to attract neutrophils to airway spaces and to other infection sites (5, 6). Neutrophils, which are capable of killing S. pneumoniae through a number of mechanisms, are thought to be ultimately responsible for clearing up infection (7). The importance of TLRs in antipneumococcal host defenses is underlined by the strong association between defects in the TLR–NF-κB signaling pathway and invasive S. pneumoniae infections in children (8). For example, approximately half of the patients with MyD88 or IRAK-4 deficiency are affected by one or more episodes of invasive S. pneumoniae infection (9). Similarly, mice lacking MyD88 (10, 11) or IRAK-4 (12) are extremely sensitive to pneumococcal infection and die in a few days under conditions resulting in survival of all control wild-type (WT) mice. Despite the importance of the MyD88/IRAK-4 pathway in antipneumococcal defenses, the exact role of individual TLRs or other MyD88-dependent receptors, such as IL-1/18R, is presently unclear.

Since TLR2 has been classically considered the most important receptor involved in the recognition of Gram-positive bacteria (13, 14), several studies have addressed its role in experimental pneumococcal infections in mice. Animals lacking TLR2 displayed modestly decreased lung inflammation early in the course of pneumococcal pneumonitis but were fully capable of controlling infection, and their mortality did not differ from that of wild-type controls (15, 16).

Similarly, TLR2 was not required for host defenses against postinfluenza pneumococcal pneumonia (17). However, TLR2 might play a role in potentiating cytokine responses to S. pneumoniae under some circumstances. Spleen macrophages and dendritic cells lacking TLR2 displayed a reduced ability to produce TNF-α and IL-1β when stimulated *in vitro* with heat-killed pneumococci (18). In contrast, TLR2^−/−^ spleen cells from mice injected intraperitoneally with heat-killed bacteria showed unaltered *in vivo* cytokine expression, although they had reduced ability to mount type 1 humoral adaptive responses (19). Interestingly, TLR2 was found to be required for effective innate antipneumococcal responses in the brain in models involving intracranial inoculation of bacteria (20–22). Only one study examined the role of TLR9, which was shown to trigger activation of resident macrophages and early bacterial clearance, but not proinflammatory cytokine or chemokine production, in a model of pneumococcal pneumonia (23).

The role of TLR4 in pneumococcal infection is more controversial. Pneumolysin, a cytolytic toxin from S. pneumoniae, was previously reported to exert proinflammatory and proapoptotic activities by TLR4-dependent (24, 25) and TLR-4 independent (26) mechanisms. Moreover, this receptor was reported to play a central role in promoting nasopharyngeal and pulmonary host defenses against S. pneumoniae (24). Other reports, however, documented limited (27) or no (23, 28) effects of TLR4 deficiency in host resistance to pneumococci. Collectively, these studies indicate that the activation of TLR2, TLR4, or TLR9 has by itself a limited role in antipneumococcal host defenses and cannot explain the severe phenotype observed in MyD88-deficient mice. There is clearly a need to better assess the relative importance of the various TLRs in host defenses, in order to develop new therapeutic agents capable of enhancing bactericidal host responses during infections caused by antibiotic-resistant strains (29). In the present study, we took advantage of the availability of several mouse strains with single or multiple defects in TLRs or TLR-dependent signaling molecules to directly compare the roles of a wide range of these receptors in the same model of pneumococcal disease. We found that the RNA-sensing receptors TLR7 and TLR13 play a previously unrecognized role in the immune detection of these bacteria. Moreover, the simultaneous absence of TLR7, TLR9, and TLR13 was associated with extreme susceptibility to infection, due to the inability of resident macrophages to produce chemokines and recruit neutrophils to infection sites.

## RESULTS

### Mice with defective UNC93B1 function are highly susceptible to pneumococcal disease.

**FIG 1:**
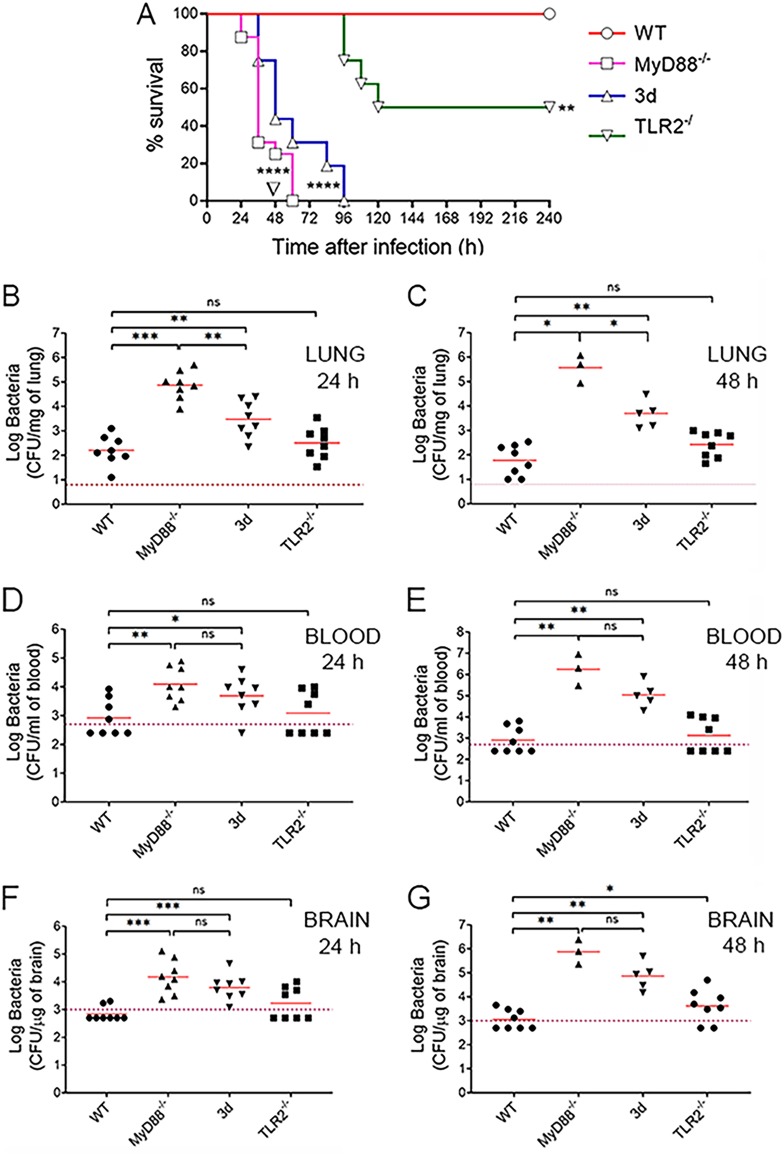
Mice with defective UNC93B1 function are highly susceptible to pneumococcal infection. (A) Survival of WT, 3d, MyD88^−/−^, or TLR2^−/−^ mice after i.n. challenge with 5.5 × 10^7^ CFU of S. pneumoniae strain D39. Shown are cumulative data from two experiments, each involving 8 animals per group. ****, *P* < 0.0001 versus WT mice, as determined by Kaplan-Meier analysis. ∇, *P* < 0.05 versus 3d, as determined by Kaplan-Meier analysis. (B to G) Log CFU numbers in the lung (B and C), blood (D and E), and brain (F and G) of WT, 3d, MyD88^−/−^, or TLR2^−/−^ mice at 24 (B, D, and F) and 48 (C, E, and G) h after i.n. infection. Horizontal bars indicate mean values. The dashed lines indicate the limit of detection of the test. Each determination was conducted on a different animal in the course of two experiments, each involving 4 animals per group. *, *P* < 0.05; **, *P* < 0.01; ***, *P* < 0.001 versus WT mice, determined by the Wilcoxon test; ns, not significant.

Little is known of the role of nucleic acid-sensing TLRs in pneumococcal recognition. To investigate this, we used mice (named “3d”) with a loss-of-function mutation in the chaperone protein UNC93B1, which allows TLR trafficking from the endoplasmic reticulum (30). The 3d mutation prevents localization of nucleic acid-sensing TLRs to the endosomes and, therefore, their signaling function. The phenotype of 3d mice was analyzed in an infection model in which bacteria are inoculated by a respiratory route, replicate in the lung, and spread hematogenously to the brain. In each experiment, we included for comparison mice lacking MyD88 or TLR2, each of which has been previously studied in similar models of pneumococcal infection. To mimic the host-pathogen interactions that occur in most natural infections, mice were given a dose of bacteria (5.5 × 10^7^) that is insufficient, by itself, to cause death in wild-type animals. Under these conditions, all mice lacking MyD88 died in less than 3 days, while none of the wild-type animals showed signs of disease until the end of the experiment (Fig. 1A). Strikingly, 3d mice also rapidly succumbed to infection, and their survival time was only slightly different from that of MyD88-defective animals. Mice lacking TLR2 survived infection for 4 days in good condition, but after this time, half of them showed signs of irreversible disease and were humanely euthanized (Fig. 1A). These animals showed the presence of high numbers of bacteria in the brain (not shown), suggesting encephalitis as the cause of their illness. To confirm that the phenotype of the immune-defective mice was due to a reduced ability to control *in vivo* replication of pneumococci, we next measured bacterial burden in the organs of infected animals.

Pneumococci were detected in the lungs of all wild-type mice at both 24 and 48 h after challenge. However, these animals were at least partially able to contain infection in the lung, and only half of them showed the presence of bacteria in the blood and brain (Fig. 1B to G). In contrast, pneumococci were present in the blood and brain of all 3d mice. Moreover, in 3d mice bacterial burden was 1 to 2 orders of magnitude higher than that of wild-type animals and only moderately lower than that of MyD88^−/−^ mice, which are known to be extremely susceptible to infection (Fig. 1B to G). These data indicate that the increased lethality of 3d mice is caused by their diminished ability to control bacterial growth in different organs.

### Mice with single defects in TLR7, TLR9, or TLR13 have moderately increased susceptibility to pneumococcal encephalitis.

**FIG 2:**
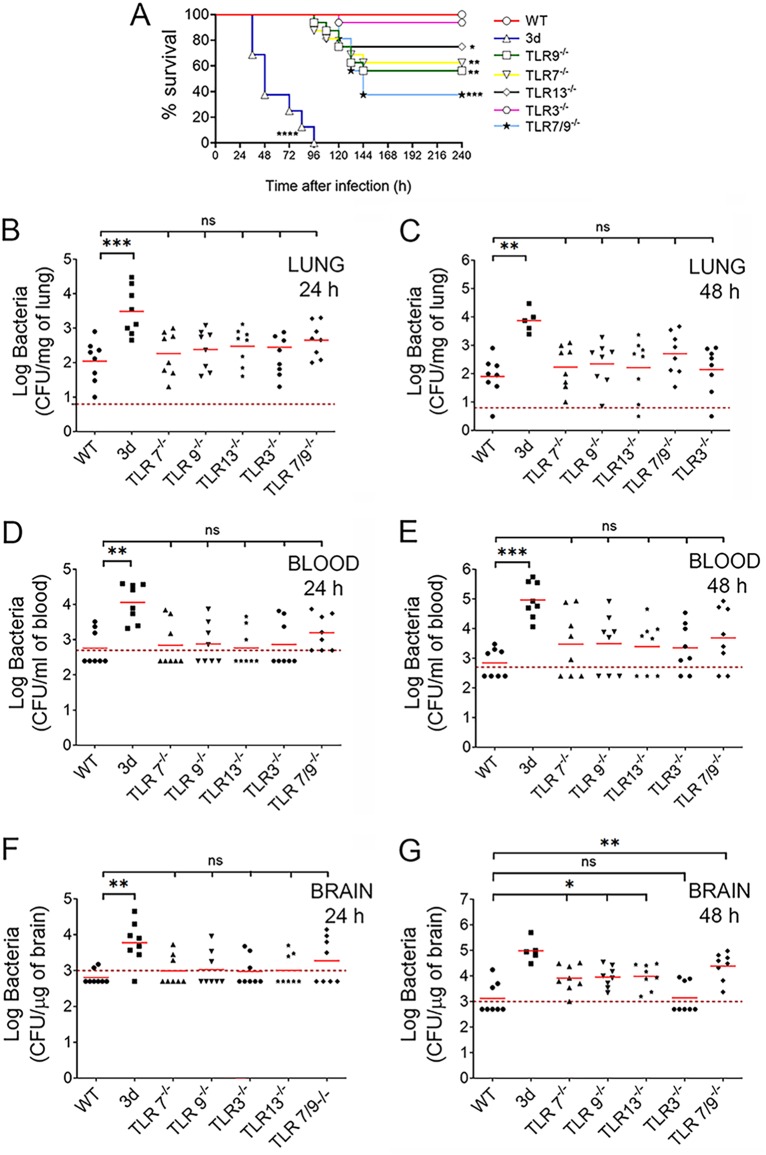
Mice with single defects in TLR7, TLR9, or TLR13 have moderately impaired antipneumococcal defenses. (A) Survival of WT mice, 3d mice, or mice lacking single (TLR3, TLR7, TLR9, or TLR13) or multiple (TLR7/9) endosomal TLRs after i.n. challenge with 5.5 × 10^7^ CFU/mouse. Data shown are the cumulative results from two experiments, each involving 8 animals per group. *, *P* < 0.05; **, *P* < 0.01; ***, *P* < 0.001; ****, *P* < 0.0001 versus WT mice, as determined by Kaplan-Meier survival plots. (B to G) Colony counts in the lung (B and C), blood (D and E), and brain (F and G) of the indicated mouse strains at 24 (B, D, and F) and 48 (C, E, and G) h after i.n. infection. Horizontal bars indicate mean values. The dashed lines indicate the limit of detection of the test. Each determination was conducted on a different animal in the course of two experiments, each involving 4 animals per group. *, *P* < 0.05; **, *P* < 0.01; ***, *P* < 0.001 versus WT mice, as determined by Wilcoxon test; ns, not significant.

The extreme susceptibility to infection of 3d mice suggested the involvement of one or more endosomal TLRs in antipneumococcal defenses. To identify relevant TLRs, we tested mice lacking TLR3, TLR7, TLR9, or TLR13, as well as TLR7/9^−/−^ double knockout (KO) mice, for their ability to control pneumococcal infection. Mice with the 3d mutation were included in each experiment for comparison. As expected, all wild-type animals survived until the end of the experiment, while all 3d mice succumbed to infection within 4 days after challenge (Fig. 2A). Only a fraction (approximately 30 to 45%) of TLR7, TLR9, or TLR13 KO mice died, and these deaths were significantly delayed relative to those of 3d mice. Double TLR7/9 deficiency resulted in additive lethality, with 62% of the doubly deficient animals succumbing to infection between 4 and 6 days after challenge (Fig. 2A). Except for TLR3^−/−^ animals, whose survival was similar to that of WT mice (Fig. 2A), increased bacterial burden was detected in the brain, but not in the lung or blood, of all TLR-defective mice (Fig. 2B to G). Encephalitis was the likely cause of death in these animals, since high CFU numbers were found in the brains of diseased animals (not shown). Collectively, these data indicate that mice with single defects in TLR7, TLR9, or TLR13 have moderately impaired antipneumococcal defenses in the brain resulting in late-onset lethality.

### The simultaneous absence of TLR7, TLR9, and TLR13 recapitulates the phenotype of 3d mice.

**FIG 3:**
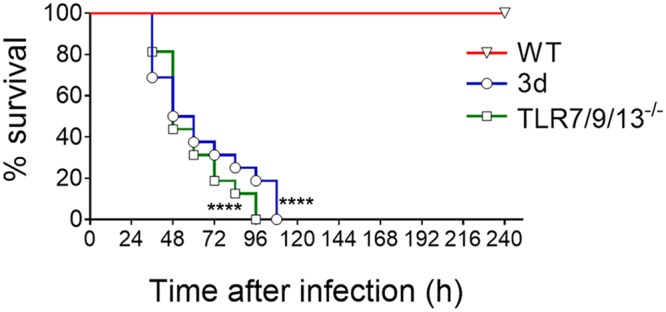
Effects of the simultaneous absence of TLR7, TLR9, and TLR13 on survival of pneumococcal infection. Kaplan-Meier analysis of survival rates in WT, 3d, or TLR7/9/13 triple KO mice after i.n. challenge with 5.5 × 10^7^ bacteria. Data are the cumulative results from two experiments, each involving 8 animals per group. ****, *P* < 0.0001 versus WT mice, as determined by Kaplan-Meier analysis.

**FIG 4:**
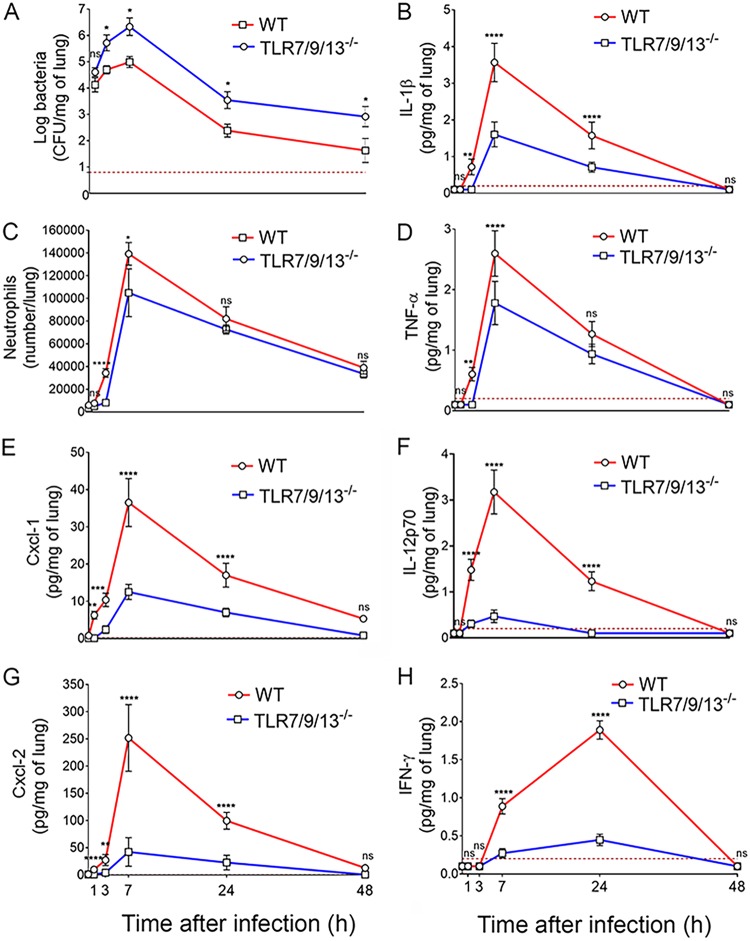
Effect of the simultaneous absence of TLR7, TLR9, or TLR13 on neutrophil recruitment and cytokine production in the lung during pneumococcal infection. Kinetics of bacterial burden (A), neutrophil counts (C), and cytokine concentrations (B and D to H) in lung homogenates after i.n. challenge with S. pneumoniae (5.5 × 10^7^ CFU/mouse). Lung homogenates were collected at different times after infection (1, 3, 7, 24, and 48 h). The dashed lines indicate the limit of detection of the test. Shown are cumulative data from two experiments, each involving 4 animals per group. *, *P* < 0.05; **, *P* < 0.01; ***, *P* < 0.001; ****, *P* < 0.0001 versus WT mice, determined by Wilcoxon test (A) and Student’s *t* test (B to H); ns, not significant.

**FIG 5:**
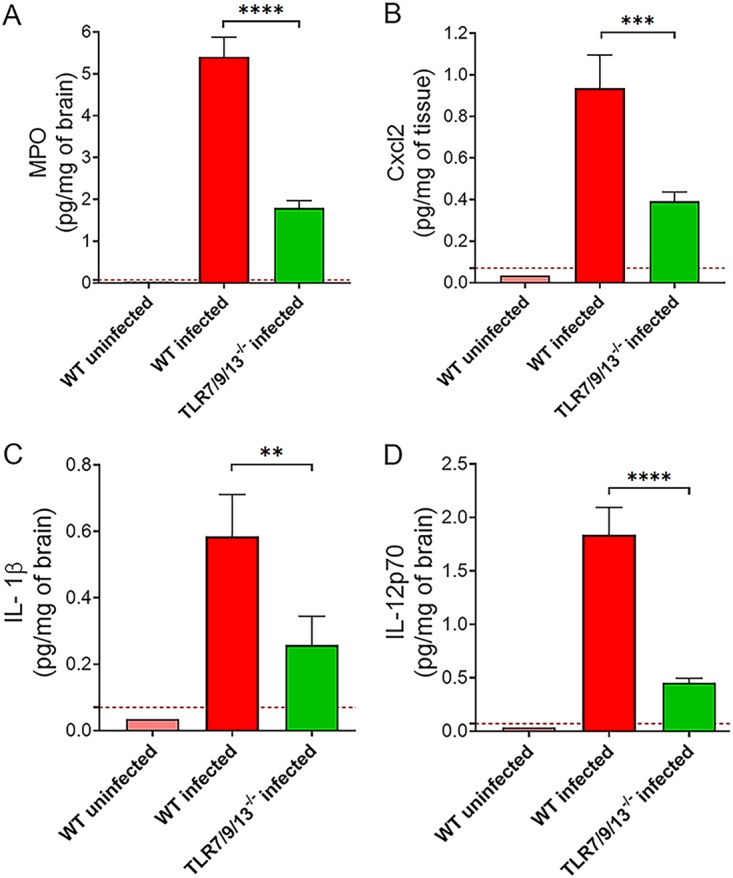
Effect of the simultaneous absence of TLR7, TLR9, and TLR13 on cytokine production in the brain. MPO (A), Cxcl2 (B), IL-1β (C), and IL-12p70 (D) protein levels in brain homogenates from WT and TLR7/9/13-deficient mice were measured at 48 h after i.n. infection with 5.5 × 10^7^ CFU of S. pneumoniae. The dashed lines indicate the limit of detection of the test. Data are expressed as the means + SD from four observations, each conducted on a different animal, during the course of one experiment. **, *P* < 0.01; ***, *P* < 0.001; ****, *P* < 0.0001 versus WT mice, determined by Student’s *t* test.

To ascertain if the phenotype of 3d mice could be recapitulated by the simultaneous absence of multiple endosomal receptors, we developed TLR7/9/13 triple KO mice and tested them for susceptibility to pneumococcal infection. Figure 3 shows that triple TLR7/9/13-deficient mice all succumbed to infection within 4 days, thus showing the same degree of susceptibility to infection displayed by 3d mice. We next determined the kinetics of cytokine production, neutrophil recruitment, and bacterial growth in lung homogenates from the triple KO animals. As expected, bacterial numbers were significantly higher at all times in the immune-deficient mice relative to wild-type ones. In both groups of animals, bacterial burden increased early after challenge, peaking at 7 h and declining thereafter (Fig. 4A). This decrease in CFU was coincident in timing with neutrophil influx, which was markedly reduced in triple KO animals early after challenge, as evidenced by reduced neutrophil numbers (Fig. 4C) and myeloperoxidase (MPO) levels (see Fig. S1A in the supplemental material) in lung homogenates. The neutrophil-attracting chemokines Cxcl1 and Cxcl2 were the first inflammatory mediators to be produced during infection, being already detectable at 1 h postchallenge in wild-type animals (Fig. 4E and G). Strikingly, the levels of these chemokines were lower in the triple KO mice at all times during infection, despite the presence of much higher bacterial numbers. In addition, while the levels of IL-1β and TNF-α were moderately decreased in triple KO mice, elevations in IL-12p70, and subsequently in IFN-γ, were virtually abrogated relative to wild-type animals (Fig. 4B to H). Similar defects in neutrophil recruitment and proinflammatory cytokine production during pneumococcal infection were detected in 3d mice (data not shown). Next, we measured the concentrations of chemokines and cytokines in brain homogenates obtained from triple KO and wild-type animals at 48 h after challenge. These brain homogenates were also tested for concentrations of myeloperoxidase, a marker that was found to correlate closely with neutrophil numbers in the lung in the previous experiments (compare Fig. 4C and Fig. S1A). The concentrations of Cxcl2, IL-1β, IL-12p70, and myeloperoxidase were all significantly decreased in brain homogenates obtained from the immune-defective animals (Fig. 5), mirroring the results obtained in the lungs. Collectively, these data indicate that the reduced ability of 3d and TLR7/9/13 triple KO mice to control infection is associated with reduced early neutrophil influx in the lung and brain and with markedly impaired production of neutrophil chemokines and proinflammatory cytokines, particularly IL-12p70 and gamma interferon (IFN-γ).

10.1128/mBio.00415-20.1 FIG S1 MPO production in the lungs of infected mice and ROS production by macrophages stimulated with S. pneumoniae. (A) MPO levels in lung homogenates from WT and TLR7/9/13-deficient mice measured at different times (1, 3, 7, 24, and 48 h) after i.n. infection with 5.5 × 10^7^ CFU of S. pneumoniae D39. The dashed lines indicate the limit of detection of the test. Data are expressed as the means ± SD from three observations, each conducted on a different animal, during the course of one experiment. (B) Delta median fluorescence intensities (ΔMFI) of macrophages stimulated with graded doses (MOI of 50, 100, and 200) of S. pneumoniae D39 and stained with CellROX Deep Red. Data are expressed as the means ± SD from three duplicate observations, each conducted during a different experiment. *, *P* < 0.05; **, *P* < 0.01 versus WT mice, determined by Student’s *t* test. Download FIG S1, PDF file, 0.1 MB. Copyright © 2020 Famà et al. 2020 Famà et al. This content is distributed under the terms of the Creative Commons Attribution 4.0 International license.

### Endosomal TLRs are required for chemokine and cytokine responses to pneumococci in resident macrophages.

**FIG 6:**
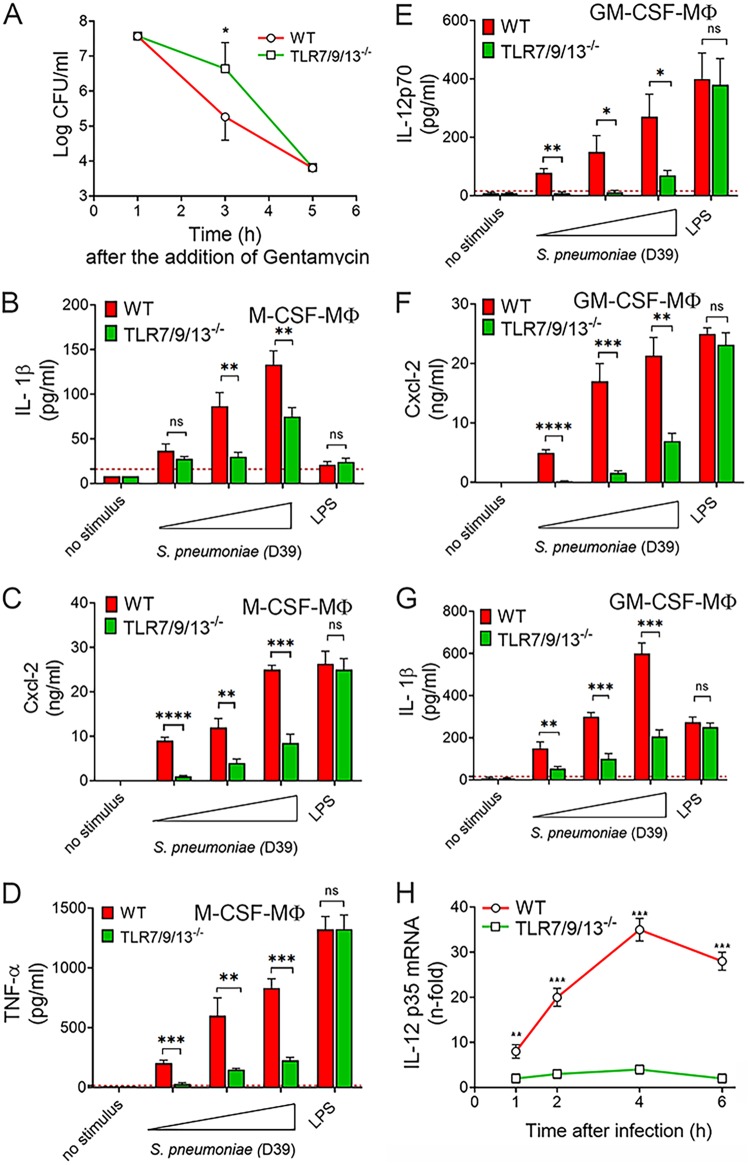
Endosomal TLRs are required for chemokine and cytokine responses to pneumococci in BMDMs. (A) Bacterial killing assay in M-CSF-polarized BMDMs from WT and TLR7/9/13 triple KO mice. BMDMs (5 × 10^5^) were incubated with S. pneumoniae (MOI of 20) for 1 h at 37°C with 5% CO_2_. Extracellular bacteria were then killed through the addition of medium containing gentamicin (100 μg/ml), and cultures were subsequently further incubated for 1, 3, and 5 h at 37°C with 5% CO_2_ before determination of CFU in cell lysates. Means ± SD from five independent experiments conducted in duplicate are shown. *, *P* < 0.05 versus WT mice as determined by Wilcoxon test. (B to D) IL-1β, Cxcl2, and TNF-α concentrations in 24-h culture supernatants of M-CSF-polarized BMDMs stimulated with increasing amounts (MOI of 5, 10, and 20) of bacteria or LPS (0.1 μg/ml). (E to G) IL-12p70, Cxcl2, and IL-1β protein levels in 24-h culture supernatants of GM-CSF-polarized BMDMs stimulated with increasing amounts (MOI of 5, 10, and 20) of bacteria or LPS (0.1 μg/ml). (H) RT-qPCR assessment of IL-12p35 mRNA levels in GM-CSF-differentiated BMDMs at different times after stimulation with S. pneumoniae (MOI of 10). The dashed lines indicate the limit of detection of the test. Means ± SD from three independent experiments conducted in duplicate are shown. *, *P* < 0.05; **, *P* < 0.01; ***, *P* < 0.001; ****, *P* < 0.0001 versus WT mice, determined by Student’s *t* test; ns, not significant.

Macrophages have a central role in killing pathogens and in orchestrating antibacterial defenses through the release of cytokines and chemokines. In view of the severe reduction in *in vivo* production of chemokines and cytokines observed in TLR7/9/13 triple KO mice during infection, it was of interest to analyze the ability of macrophages from these mice to respond to pneumococci. Therefore, we obtained bone marrow-derived, macrophage colony-stimulating factor (M-CSF)-polarized macrophages (BMDMs) from immune-deficient mice and tested them for bactericidal activity and mediator production in response to graded pneumococcal doses. Under these conditions, cells remained viable for the entire duration of the assay, as shown by persistence of low levels of lactate dehydrogenase, a sensitive indicator of cell toxicity, and similar data were obtained with neutrophils (data not shown). BMDMs from TLR7/9/13 triple KO mice were as capable of ingesting pneumococci as wild-type animals, while intracellular killing was only slightly delayed (Fig. 6A), in association with decreased production of reactive oxygen species (Fig. S1B). Moreover, triple KO cells were impaired in the production of Cxcl1/2, TNF-α, and IL-1β, compared with control cells, particularly when using low bacterial doses as a stimulus (Fig. 6B to D and Fig. S2A). As expected, BMDMs from the immune-deficient mice were fully capable of responding to lipopolysaccharide (LPS), a TLR4 ligand, used as a positive control.

10.1128/mBio.00415-20.2 FIG S2 Cxcl1 protein levels in BMDMs from TLR7/9/13 triple KO. M-CSF (A)- or GM-CSF (B)-polarized BMDMs were stimulated with increasing doses of S. pneumoniae (MOI of 5, 10, and 20) or LPS (0.1 μg/ml), and Cxcl1 levels were measured in 24-h culture supernatants. Means + SD from three independent experiments conducted in duplicate. **, *P* < 0.01; ***, *P* < 0.001; ****, *P* < 0.0001 versus wild-type mice, determined by Student’s *t* test. Download FIG S2, PDF file, 0.1 MB. Copyright © 2020 Famà et al. 2020 Famà et al. This content is distributed under the terms of the Creative Commons Attribution 4.0 International license.

In addition to M-CSF, granulocyte-macrophage colony-stimulating factor (GM-CSF) plays a crucial role in macrophage maturation, particularly in the differentiation of alveolar macrophages (31). To determine whether the polarization status of macrophages influences the role of endosomal receptors, we also used GM-CSF-differentiated bone marrow-derived cells. GM-CSF-polarized cells were even more dependent on TLR7/9/13 for cytokine production in response to pneumococci than M-CSF-polarized ones (Fig. 6E to G and Fig. S2B). Remarkably, GM-CSF-polarized cells produced high levels of IL-12p70, and these responses were almost completely abrogated in triple KO cells (Fig. 6E). Next, it was of interest to ascertain whether impaired transcription of the IL-12-encoding genes (p35 and p40) could account for the reduced IL-12p70 response observed in TLR7/9/13 triple KO GM-CSF-polarized macrophages. In wild-type cells, both IL-12p35 and IL-12p40 mRNA levels significantly increased after stimulation with pneumococci, peaking at 4 h (Fig. 6H and Fig. S3A). The IL-12p35 mRNA elevations were almost completely abrogated in triple KO cells, while the IL-12p40 RNA response was only moderately impaired, suggesting that the defect in IL-12p70 production observed in the absence of TLR7/9/13 is due to a selective decrease in transcription of the IL-12p35 gene. Lack of isolated TLRs resulted in moderate (TLR7^−/−^, TLR13^−/−^, and TLR7/9 double KO) or no (TLR2^−/−^ and TLR9^−/−^) decrease in IL-12p70 production (Fig. S3B).

10.1128/mBio.00415-20.3 FIG S3 IL-12 responses to stimulation with pneumococci in GM-CSF-polarized BMDMs. (A) RT-qPCR assessment of IL-12p40 mRNA levels in GM-CSF-differentiated BMDMs at different times after stimulation with S. pneumoniae (MOI of 10). (B) IL-12p70 protein levels in 24-h culture supernatants of GM-CSF-polarized BMDMs from mice lacking single or multiple TLRs after stimulation with S. pneumoniae (MOI of 10). The dashed lines indicate the limit of detection of the test. Means ± SD from three independent experiments conducted in duplicate. *, *P* < 0.05; **, *P* < 0.01; ***, *P* < 0.001; ****, *P* < 0.0001 versus WT mice, as determined by Student’s *t* test (A) or one-way analysis of variance (B). Download FIG S3, PDF file, 0.1 MB. Copyright © 2020 Famà et al. 2020 Famà et al. This content is distributed under the terms of the Creative Commons Attribution 4.0 International license.

**FIG 7:**
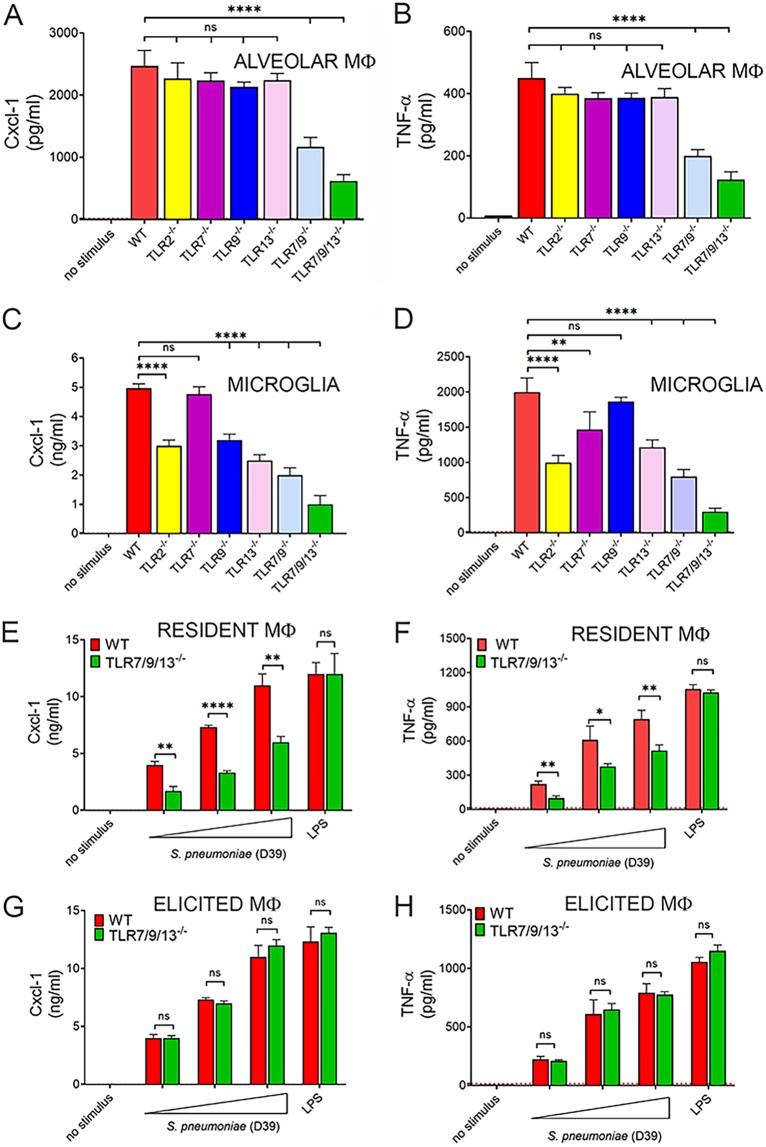
Role of endosomal TLRs in cytokine responses of macrophages from different tissues. Alveolar macrophages (A and B) and microglia (C and D) from wild-type mice (WT) or mice lacking single or multiple endosomal TLRs were stimulated with S. pneumoniae (MOI of 10), and cytokine concentrations were measured in 24-h culture supernatants. Resident peritoneal macrophages (E and F) and thioglycolate-elicited peritoneal macrophages (G and H) were obtained from the peritoneal cavity of WT or TLR7/9/13 triple KO mice and stimulated with increasing MOI (5, 10, and 20) of S. pneumoniae or LPS (0.1 μg/ml). TNF-α and Cxcl1 levels were measured in 24-h culture supernatants. The dashed lines indicate the limit of detection of the test. Means + SD from three independent experiments conducted in duplicate. *, *P* < 0.05; **, *P* < 0.01; ****, *P* < 0.0001 versus WT mice, determined by one-way analysis of variance (A to D) or Student’s *t* test (E to H); ns, not significant.

In further experiments, it was of interest to ascertain whether endosomal receptors play a role in cytokine responses to other bacteria, in addition to the pneumococcal serotype 2 D39 strain used throughout this study. Figure S4 shows that this was indeed the case, since reduced levels of TNF-α, Cxcl2, and IL-12p70 were measured in M-CSF- and GM-CSF-polarized macrophages after stimulation with group A streptococcus, Escherichia coli, and the serotype 4 TIGR4 pneumococcal strain. Macrophages residing in different anatomical sites display different surface markers, functional activities, and immune receptor usage (32). Since the lung and the central nervous system are important targets of invasive pneumococcal infection, we next examined resident alveolar macrophages (AM) and microglia for their ability to recognize pneumococci via endosomal TLRs. Cytokine concentrations were significantly lower in cultures of TLR7/9/13 triple KO AM or microglia stimulated with pneumococci, compared with wild-type mice (Fig. 7A to D). Moreover, moderate reductions in Cxl1 and TNF-α levels were observed in microglia, but not AM, lacking single TLRs, including TLR2, TLR7, TLR9, or TLR13 (Fig. 7A to D). Because pneumococci can interact with phagocytes differing in type and origin (e.g., resident versus inflammatory), we also examined resident and thioglycolate-elicited peritoneal macrophages. The role of endosomal receptors in resident peritoneal macrophages was similar to that previously observed in BMDMs, AM, and microglia (Fig. 7E and F). In marked contrast, however, thioglycolate-elicited peritoneal macrophages from TLR7/9/13 triple KO or 3d mice were fully capable of mounting high-level cytokine responses (Fig. 7G and H). Taken together, these data indicated that the simultaneous absence of the nucleic acid-sensing receptors TLR7, TLR9, and TLR13 markedly impairs the ability of macrophages residing in different tissues to respond to pneumococci but has no effect on responses elicited in inflammatory macrophages.

10.1128/mBio.00415-20.4 FIG S4 Effect of the simultaneous absence of TLR7, TLR9, and TLR13 on cytokine production in macrophages. TNF-α, Cxcl2, and IL-12p70 concentrations in 24-h culture supernatants of M-CSF (A and C)- or GM-CSF (B and D)-polarized BMDMs stimulated with an MOI of 10 of S. pneumoniae TIGR4 strain, GAS strain 3348, or E. coli strain K1 E-R8. ns, no stimulus. The dashed lines indicate the limit of detection of the test. Data are expressed as the means + SD from three duplicate observations, each conducted during a different experiment. *, *P* < 0.05; **, *P* < 0.01; ***, *P* < 0.001 versus WT mice, determined by Student’s *t* test. Download FIG S4, PDF file, 0.1 MB. Copyright © 2020 Famà et al. 2020 Famà et al. This content is distributed under the terms of the Creative Commons Attribution 4.0 International license.

## DISCUSSION

The identification of receptors involved in immune sensing of S. pneumoniae has attracted considerable attention over the last 2 decades, since such knowledge is crucial to understand the pathogenesis of pneumococcal disease and to develop much-needed alternative strategies to treat infections by antibiotic-resistant strains (33). An essential role of TLRs in antipneumococcal host defenses is suggested by the extreme susceptibility of children with MyD88/IRAK-4 deficiency to invasive S. pneumoniae infections (9, 34), and a similar phenotype is also observed in mice (10–12). However, the individual MyD88/IRAK-dependent receptors responsible for this profound defect in host defenses have not been identified yet. Many investigations have previously dealt with the role of TLR2 and TLR4, which are predominantly located on the cell surface (13). In contrast, the functional importance of nucleic acid-sensing endosomal TLRs has not been investigated in the context of antipneumococcal host defenses, with the exception of two studies focusing on TLR3 (35) and TLR9 (23), respectively. An important finding of the present study is that the hypersusceptibility of MyD88-deficient mice to S. pneumoniae infection can be largely recapitulated by the simultaneous absence of the endosomal receptors TLR7, TLR9, and TLR13. While single TLR deficiencies were associated with unaltered ability to clear lung infection, the combined lack of TLR7, TLR9, and TLR13 resulted in uncontrolled bacterial growth, hematogenous dissemination, and early lethality. Our data point to a dominant role of nucleic acid-sensing pathways in innate immune recognition of S. pneumoniae and indicate that surface TLRs, such as TLR2 or TLR4, cannot compensate for defects in endosomal TLR signaling.

The simultaneous absence of TLR3/7/9 was associated with a significant reduction in early influx into the lung of neutrophils, which are primarily responsible for clearing pneumococcal infection (7, 36). Defective recruitment of these cells was likely a consequence of decreased production of neutrophil chemoattractants, such as Cxcl1/2, IL-1β, and TNF-α (37). Moreover, release of IL-12p70 and IFN-γ, which are also involved in phagocyte recruitment and potentiation of phagocytic killing (38–41), was almost completely abrogated in the triple KO mice. Thus, the mechanisms underlying the hypersusceptibility of TLR7/9/13 triple KO mice to pneumonia are similar to those previously identified in MyD88-deficient mice, in which chemokine production and neutrophil recruitment to the lung were severely impaired (11). In the present study, the defects in chemokine and cytokine production observed *in vivo* were mirrored *in vitro* by impaired release of these mediators by AM and other resident macrophages after stimulation with pneumococci. However, since both myeloid and epithelial cells contribute to MyD88-dependent responses to these bacteria (11), further studies are needed to investigate the role of endosomal TLRs in airway epithelial cells.

We have used here a sensitive pneumococcal infection model, in which wild-type mice develop transient pneumonitis. Under these conditions, bacteria reach the blood and the brain in approximately half of the animals, but the infection is eventually cleared without signs of disease. Using this model, mice with single deletions in specific TLRs, such as TLR2, TLR7, TLR9, or TLR13 were fully capable of controlling bacterial replication in the lung, but 30 to 45% of them developed encephalitis late during infection. Notably, we have been able to link this increased susceptibility to encephalitis with defective production of neutrophil chemokines and proinflammatory cytokines by microglia cells in response to pneumococci. Indeed, while single TLRs were completely dispensable for mediator production in alveolar, peritoneal, and bone marrow macrophages, TLR2, TLR7, TLR9, and TLR13 each made a moderate but significant contribution to antipneumococcal responses in microglia. Thus, control of pneumococcal infection in the central nervous system apparently depends on the additive, cooperative activity of multiple cell surface (TLR2) and endosomal (TLR7, TLR9, and TLR13) receptors.

In the present study, some cytokine responses were more affected than others by the absence of endosomal TLRs. For example, the IL-12p70 and Cxcl2 responses to lung infection were almost completely abrogated in TLR7/9/13 triple KO mice, while the TNF-α response was only moderately reduced. This may be related to the fact that selected genes, such as the IL-12p35 gene, are specifically targeted by the activation of TLR7/9 via the interferon regulatory factor 1 (IRF1) transcription factor (42–44). Studies are in progress to ascertain if the same holds true for Cxcl1/2 genes and if IRF1 is also targeted by TLR13, in addition to TLR7/9. Collectively, our data indicate that the origin and tissue microenvironment of macrophages can profoundly impact the contribution of individual TLRs to overall antipneumococcal defenses. These data extend previous observations on the differential roles of endosomal TLRs in the responses to group B streptococci (GBS) of macrophages isolated from different tissues (45). In this context, it seems also notable that resident, but not inflammatory, macrophages required here endosomal TLRs for antipneumococcal responses despite the fact that both cell types strictly depended on MyD88 for these activities. It is possible that the more redundant recognition system used by inflammatory macrophages relative to resident ones is linked to the upregulation of membrane TLRs and other MyD88-dependent receptors in response to inflammatory stimuli (46), but further studies are required to test this hypothesis. Our data showing normal antipneumococcal lung defenses in TLR2 and TLR3 knockout mice confirm those of previous studies (15, 16, 35). In a previous investigation, it was found that mice lacking TLR9 had slightly reduced antipneumococcal defenses in the lung (23), while we observed here normal lung defenses in these animals. This small discrepancy might be linked to differences in the experimental models and bacterial strains used.

Our data on the involvement of the RNA-sensing receptors TLR7 and TLR13 in immune detection of S. pneumoniae are in agreement with recent studies using other bacterial pathogens. Demonstration of the important role of prokaryotic RNA as a target in bacterial detection came first in the context of responses to group B streptococci (43) and was later confirmed and extended using group A streptococci and other Gram-positive bacteria (45, 47, 48). Moreover, endosomal TLRs may play a role in recognition of Gram-negative bacteria, in addition to Gram-positive ones, as suggested by our observations that cytokine responses to E. coli are impaired in TLR7/9/13 triple KO macrophages. However, further studies using various bacterial species are required to better analyze the role of endosomal TLRs in the recognition of Gram-negative pathogens. Our data using mouse macrophages parallel recent findings using human cells on the importance of RNA-sensing receptors in antipneumococcal responses, specifically TLR8 in primary monocyte-derived macrophages (49) and TLR3 in dendritic cells (35). Clearly, RNA-sensing TLRs and their cognate target sequences differ in humans and mice, since murine TLR8 is largely nonfunctional (50), while TLR13 is absent in humans. However, the general strategy of focusing on prokaryotic-type RNA for bacterial sensing seems evolutionarily conserved. For example, in human monocyte-derived macrophages, TLR8 is generally responsible for the recognition of bacterial species that stimulate TLR13 in mouse cells (45, 49, 51–53). However, it is possible that other RNA-sensing receptors (such as TLR7 and TLR3) have a prominent role in other types of human cells, and studies are under way to clarify this point.

Collectively, our present and previous data highlight the presence in phagolysosomes of a multimodal, TLR-based bacterial detection system, whereby each receptor recognizes a distinct type of nucleic acid released from ingested bacteria (43, 53). These endosomal receptors function in an integrated, additive fashion and can compensate to a certain extent for the absence of each other, depending on the origin and microenvironment of the immune cells involved. Such a redundancy seems crucial to prevent pathogens from escaping immune recognition and is achieved by the ability of MyD88-dependent endosomal TLRs to activate overlapping gene induction programs as shown, for example, in the case of TLR7 and TLR13 (54). Our data suggest that potentiation of endosomal receptor function could represent a viable strategy for improving the outcome of severe S. pneumoniae infections caused by antibiotic-resistant strains.

## MATERIALS AND METHODS

### Mouse and bacterial strains.

C57BL/6 wild-type mice were purchased from Charles River Laboratories (Charles River, Calco, Italy). Mice lacking MyD88, TLR2, TLR3, TLR7, or TLR9 were donated by Shizuo Akira (Osaka University, Japan). Heterozygous TLR13^−/+^ mice were generated in collaboration with the University of California Davis Mouse Biology Program (MBP) (www.mousebiology.org) and provided by the KOMP Repository (www.komp.org) as previously reported (55). Subsequently, TLR13^−/−^ mice were bred in the Animal Facilities of the Department of Pathology of the University of Messina, Messina, Italy. TLR7/9^−/−^ double KO mice were donated by Stephan Bauer (University of Marburg, Germany). TLR7/9/13^−/−^ triple KO mice were generated by crossing TLR7^−/−^, TLR9^−/−^, and TLR13^−/−^ mice. 3d mutant mice, bearing the H412R mutation of UNC93B1, were obtained from Bruce Beutler (University of Texas Southwestern Medical Center, Texas). All KO mice were bred onsite on a C57BL/6J background and developed normally. All mice used in the present study were housed under specific-pathogen-free conditions in individually ventilated cages. Streptococcus pneumoniae serotype 2 strain D39 was used throughout the present study. The capsular serotype 4 S. pneumoniae strain TIGR4, group A *Streptococcus* (GAS) strain 3348, and encapsulated Escherichia coli strain K1 E-R8 were also used in selected experiments. Bacteria were grown to mid-log phase in Todd-Hewitt broth (THB; Oxoid) supplemented with 1% (vol/vol) fetal calf serum (FCS), washed three times in nonpyrogenic phosphate-buffered saline (PBS) (0.01 M phosphate, 0.15 M NaCl [pH 7.4]; Thermo Fisher Scientific), and resuspended to the appropriate concentration in PBS.

### Murine infection model.

Six-week-old female mice were anesthetized by the intraperitoneal injection of tiletamine-zolazepam (0.1 mg/mouse) and xylazine (0.16 mg/mouse) and injected intranasally (i.n.) with 10 μl of the bacterial suspension to each nostril (5.5 × 10^7^ CFU/mouse). In each experiment, the actual number of injected bacteria was determined by colony counting. Clinical conditions and lethality rates were evaluated every 12 h for 10 days after inoculation. Mice showing signs of sepsis or neurological manifestations, including persistent hunching, rough hair, continuous jumping, running in circles, decreased mobility, and lethargy, were humanely euthanized. In other experiments, mice were euthanized at 24 and 48 h after challenge and transcardially perfused with PBS (20 ml), prior to organ collection, as previously described (56). To obtain lung homogenates, the organs were minced and enzymatically digested with collagenase, as described previously (57). Briefly, lung tissue was digested for 30 min at 37°C with 1 mg/ml collagenase I (Roche) in digestion buffer (RPMI 1640 supplemented with 5% FCS, 50 IU of penicillin/ml, 50 μg of streptomycin/ml, and 30 μg/ml DNase), before filtering through a 70-μm cell strainer (EuroClone). To obtain brain homogenates, the organs were placed in gentleMACS M tubes and processed in the gentleMACS dissociation system (Miltenyi Biotec). Bacterial CFU were measured in organ homogenates by plating serial dilutions of organ homogenates on blood agar.

### Isolation of bone marrow-derived cells.

Bone marrow-derived cells were obtained from the bone marrow of WT and immune-deficient mice. Briefly, after removing the femurs and the tibias and cutting off the epiphyses of the bones, bone marrow cells were spun out from both ends of the bone shafts and resuspended in Dulbecco’s PBS without Ca^2+^ and Mg^2+^ (DPBS; EuroClone). Cell aggregates were gently dissociated by pipetting with a Pasteur pipette, and the suspension was then filtered over a 70-μm cell strainer (EuroClone) into a 50-ml Falcon tube to remove any remaining bone fragments. Marrow cells were collected by centrifugation at 400 × *g* for 15 min, resuspended to a concentration of 5 × 10^6^/ml, and cultured for 6 to 7 days in RPMI 1640 supplemented with heat-inactivated 10% FCS, penicillin (50 IU/ml), and streptomycin (50 μg/ml). Medium was supplemented with 100 ng/ml macrophage colony-stimulating factor (M-CSF) or 20 ng/ml granulocyte-macrophage colony-stimulating factor (GM-CSF) (both obtained from PeproTech) to obtain bone marrow-derived, M-CSF-polarized macrophages (BMDMs) or GM-CSF-polarized bone marrow-derived cells, respectively.

### Isolation of tissue-resident macrophages and microglia.

For the isolation of resident alveolar macrophages (AM), lungs from WT or KO mice were lavaged through an intratracheal catheter with DPBS supplemented with 1 mM EDTA. The bronchoalveolar lavage (BAL) fluids were pooled and centrifuged at 300 × *g* for 10 min. Cell pellets were resuspended to a cell concentration of 5 × 10^6^/ml in RPMI 1640 supplemented with heat-inactivated 10% FCS, penicillin (50 IU/ml), streptomycin (50 μg/ml), and 100 ng/ml M-CSF. After incubation at 37°C with 5% CO_2_ for 24 h, nonadherent cells were removed by washing three times with warm medium. Microglia were isolated as described previously (45). In brief, after the removal of meninges from the brains of neonatal (24-h-old) mice, tissues were dissociated with a pipette and washed twice by centrifugation. Cell suspensions were resuspended in RPMI 1640 supplemented with heat-inactivated 10% FCS, penicillin (50 IU/ml), and streptomycin (50 μg/ml); plated into cell culture flasks coated with 5 mg/ml poly-l-lysine (PLL); and incubated at 37°C with 5% CO_2_. After 10 days, with change of medium every 3rd day, cells were treated with 10 ng/ml M-CSF (PeproTech). Three days later, microglial cells were removed from the astrocyte bed by gently shaking the flasks for 3 h at 37°C on an orbital shaker (130 rpm) and then centrifuging them at 300 × *g* for 10 min. Harvesting was repeated after 7 days using the same procedure. Murine resident peritoneal macrophages were isolated from the peritoneal cavity of WT and KO mice by washing with ice-cold DPBS, as previously described (58). Briefly, after centrifugation, cells were resuspended in RPMI 1640 supplemented with heat-inactivated 10% FCS, penicillin (50 IU/ml), and streptomycin (50 μg/ml); seeded into the wells of 96-well cell culture plates (5 × 10^5^ per well); and incubated at 37°C with 5% CO_2_. After 24 h, nonadherent cells were removed by washing three times with warm medium. In other experiments, peritoneal macrophages were elicited by intraperitoneal (i.p.) injection of 0.5 ml of 4% thioglycolate broth 3 days before collection of peritoneal lavage fluids and macrophage isolation, as described above.

### Macrophage stimulation with pneumococci.

Isolated cells (5 × 10^5^ per well in 0.2 ml of RPMI supplemented with 10% FCS) were stimulated with PBS suspensions of S. pneumoniae at the indicated multiplicities of infection (MOI). All infections were carried out by centrifuging cell suspensions for 10 min at 400 × *g* in order to facilitate bacterium-cell interactions. After incubation for 1 h at 37°C with 5% CO_2_, penicillin (250 IU/ml) and streptomycin (250 μg/ml) were added to kill extracellular bacteria. Control wells were stimulated with Escherichia coli K-12 ultrapure LPS (InvivoGen). Cell culture supernatants were then collected at various times after infection, as indicated below, and stored at −80°C for cytokine measurements. In some experiments, extracellular lactate dehydrogenase (LDH) levels were measured in cell culture supernatants using the Thermo Scientific Pierce LDH cytotoxicity assay kit.

### Phagocytic killing assay.

For phagocytosis/killing assays, S. pneumoniae (MOI of 20) was added to 5 × 10^5^ BMDMs obtained from WT and TLR7/9/13 triple KO mice and incubated for 1 h at 37°C with 5% CO_2_. Cells were then washed three times with PBS, and extracellular bacteria were killed through the addition of medium containing gentamicin (100 μg/ml). Cells were subsequently incubated for the indicated times at 37°C with 5% CO_2_, washed, detached, and lysed with 0.025% Triton X-100 to release intracellular bacteria. Recovered bacteria were plated on blood agar plates for CFU counts.

### Cytokine and myeloperoxidase measurements.

Cytokine and intracellular myeloperoxidase (MPO) levels were measured in organ homogenates using commercial enzyme-linked immunosorbent assay (ELISA) kits. TNF-α, keratinocyte-derived chemokine (KC; Cxcl1), macrophage inflammatory protein 2 (MIP-2; Cxcl2), IL-1β, IFN-γ, IL-12p70, and MPO concentrations were determined in duplicate with DuoSet TNF-α, CXCL1/KC Quantikine, DuoSet CXCL2/MIP-2, IL-1β/IL-1F2 Quantikine, DuoSet IFN-γ, DuoSet IL-12p70, and DuoSet myeloperoxidase murine ELISA kits, respectively, according to the manufacturer’s recommendations (R&D Systems). The lower detection limits of these assays were 16, 15.6, 15.6, 12.5, 31.3, 39.1, and 250 pg/ml, respectively.

### Real-time PCR measurements of IL-12 mRNA.

Total RNA was extracted from GM-CSF-polarized macrophages (4 × 10^6^) using the RNeasy minikit (Qiagen) according to the manufacturer’s instructions. Quality and yield of RNA were determined by agarose gel electrophoresis and NanoDrop 2000 spectrophotometry (ThermoFisher Scientific), respectively. cDNA was synthesized using the Moloney murine leukemia virus reverse transcriptase kit (M-MLV RT; Invitrogen), and expression of the IL-12-encoding genes (p35 and p40) was determined by qPCR using an Applied Biosystems 7500 system by calculation of the delta-delta threshold cycle (ΔΔ*C_T_*) method normalized against the beta-actin housekeeping gene, as described previously (42).

### Flow cytometry.

Enumeration of neutrophils in lung homogenates was performed by flow cytometry on a FACS Canto II flow cytometer (BD Biosciences) using the BD Trucount kit (BD Biosciences), as previously described (56). In brief, lung homogenate cells were incubated with 0.5 μg of mouse Fc block (purified rat anti-mouse CD16/CD32 clone 2.4G2; BD Pharmingen) for 20 min before staining for 20 min with antibodies directed against Ly-6G (phycoerythrin [PE]–rat anti-mouse Ly-6G clone 1A8; BD Pharmingen) for detecting neutrophils or with an isotype control monoclonal antibody (MAb). Group B streptococcus (GBS)-induced reactive oxygen species (ROS) production by macrophages was measured using the CellROX Deep Red flow cytometry assay kit (Thermo Fisher Scientific) according to the manufacturer’s instructions. Data analysis was performed using Flowing Software 2.5.1.

### Statistical analysis.

Differences in cytokine and chemokine levels were assessed by Student’s *t* test or one-way analysis of variance (ANOVA). Survival data were analyzed by Kaplan-Meier survival plots, and differences in bacterial CFU counts were assessed by the Wilcoxon test. Differences were considered statistically significant when *P* values were less than 0.05. Statistical analyses were performed with GraphPad Prism 8.2.0 (GraphPad Software, Inc., San Diego, CA).

### Ethics statement.

All studies were performed in strict accordance with the European Union guidelines for the use of laboratory animals. The procedures were approved by the Animal Welfare Committee of the University of Messina (OPBA) and by the Ministero della Salute of Italy (permit no. 785/2018-PR).
