# Supplementary figures and images for: Nucleic Acid-Sensing Toll-Like Receptors Play a Dominant Role in Innate Immune Recognition of Pneumococci
Source: mBio. 2020 Mar 24;11(2):e00415-20. doi: 10.1128/mBio.00415-20 (PMC7157524; doi:10.1128/mBio.00415-20)

Suppl. Fig. S1

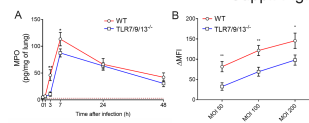

Supplement: FIG S1 [file mBio.00415-20-sf001.pdf]

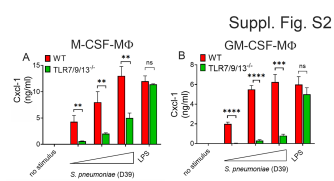

Supplement: FIG S2 [file mBio.00415-20-sf002.pdf]

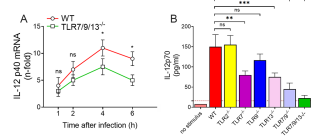

Suppl. Fig. S3

Supplement: FIG S3 [file mBio.00415-20-sf003.pdf]

Suppl. Fig. S4

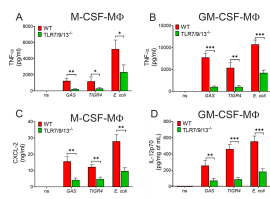

Supplement: FIG S4 [file mBio.00415-20-sf004.pdf]
